# How Do Flaviviruses Hijack Host Cell Functions by Phase Separation?

**DOI:** 10.3390/v13081479

**Published:** 2021-07-28

**Authors:** Akatsuki Saito, Maya Shofa, Hirotaka Ode, Maho Yumiya, Junki Hirano, Toru Okamoto, Shige H. Yoshimura

**Affiliations:** 1 Department of Veterinary Science, Faculty of Agriculture, University of Miyazaki, Miyazaki 889-2192, Japan; shofamaya@gmail.com; 2 Center for Animal Disease Control, University of Miyazaki, Miyazaki 889-2192, Japan; 3 Graduate School of Medicine and Veterinary Medicine, University of Miyazaki, Miyazaki 889-1692, Japan; 4 Clinical Research Center, National Hospital Organization Nagoya Medical Center, Nagoya 460-0001, Japan; hirotaka.ode@nnh.go.jp; 5 Research Institute for Microbial Diseases, Osaka University, Osaka 565-0871, Japan; yumiya@biken.osaka-u.ac.jp (M.Y.); kamati1101@biken.osaka-u.ac.jp (J.H.); 6 Center for Infectious Diseases Education and Research, Osaka University, Osaka 565-0871, Japan; 7 Laboratory of Plasma Membrane and Nuclear Signaling, Graduate School of Biostudies, Kyoto University, Kyoto 606-8501, Japan

**Keywords:** flavivirus, liquid–liquid phase separation, disordered protein, pathogenicity

## Abstract

Viral proteins interact with different sets of host cell components throughout the viral life cycle and are known to localize to the intracellular membraneless organelles (MLOs) of the host cell, where formation/dissolution is regulated by phase separation of intrinsically disordered proteins and regions (IDPs/IDRs). Viral proteins are rich in IDRs, implying that viruses utilize IDRs to regulate phase separation of the host cell organelles and augment replication by commandeering the functions of the organelles and/or sneaking into the organelles to evade the host immune response. This review aims to integrate current knowledge of the structural properties and intracellular localizations of viral IDPs to understand viral strategies in the host cell. First, the properties of viral IDRs are reviewed and similarities and differences with those of eukaryotes are described. The higher IDR content in viruses with smaller genomes suggests that IDRs are essential characteristics of viral proteins. Then, the interactions of the IDRs of flaviviruses with the MLOs of the host cell are investigated with emphasis on the viral proteins localized in the nucleoli and stress granules. Finally, the possible roles of viral IDRs in regulation of the phase separation of organelles and future possibilities for antiviral drug development are discussed.

## 1. Introduction

Viral proteins interact with different sets of host cellular components throughout the viral life cycle, beginning with entry, proceeding to replication, virion assembly, and finally exiting from the host cell surface. Since this series of events occurs in different intracellular compartments, the intracellular localization of viral proteins and genomes also plays pivotal roles in the viral life cycle. There have been a number of reports on the unique intracellular localization of viral proteins in membraneless organelles (MLOs), such as the nucleoli, stress granules (SGs), P-bodies, and other RNA-containing granules, which sometimes differ from the sites of replication and virion assembly. The formation of MLOs is mostly driven by liquid–liquid phase separation (LLPS) or condensation of disordered polypeptides (in some cases together with RNA), in which highly charged polypeptides interact with each other via promiscuous interactions to self-assemble into separated phases. In contrast to conventional “stereospecific” interactions between the solid surfaces of structured proteins, such promiscuous interactions allow for dynamic assembly/disassembly of macromolecules in the intracellular milieu and flexible responses to environmental changes. In the case of LLPS, confinement of molecules (especially enzymes and substrates) into a limited volume in the vast cytoplasmic space is considered to increase the rates and efficiency of multi-step reactions.

**Figure 1:**
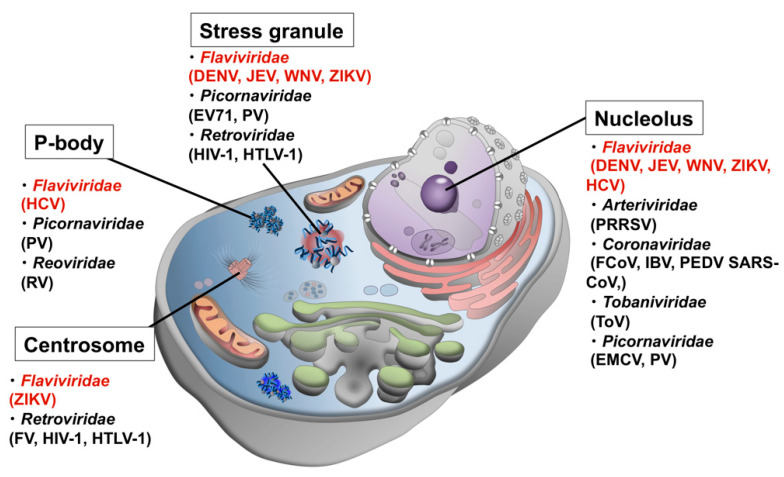
Possible interactions between host MLOs and viral proteins. Viruses in *Flaviviridae* are shown in red.

Another line of evidence from genomic and proteomic analyses using bioinformatics approaches revealed that viral proteins have high contents of disordered regions [1,2,3], which was initially interpreted as a viral strategy to efficiently use the limited genomic information by conferring multi-functionality and adaptability to a single protein. However, in the recent context of the role of disordered proteins/regions in LLPS and the formation of MLOs, it is highly probable that disordered viral proteins interact with host MLOs to either commandeer the function of the organelle to promote efficient viral replication and proliferation, or to sneak into the organelle to avoid the host cell immune response. To date, dozens of viral proteins have been found localized in MLOs of host cells (Figure 1 and Table 1).

The 70 viruses in the genus *Flavivirus* (family *Flaviviridae*), including several arthropod-borne human pathogens such as dengue virus (DENV), West Nile virus (WNV), yellow fever virus (YFV), Zika virus (ZIKV), and Japanese encephalitis virus (JEV), pose major threats to human health, mainly in tropical and subtropical countries as many of them are transmitted by mosquitoes. The clinical manifestation of flavivirus infection ranges from asymptomatic cases, mild symptoms, to life-threatening hemorrhagic fever. For example, infection with neurptropic viruses JEV and WNV can lead to death [24,25], and ZIKV can cause microcephaly in infants, as seen in the ZIKV epidemic in South America from 2015 to 2016 that resulted in a high number of confirmed cases [26]. Current situation of vaccine development targeting flaviviruses is summarized in Box 1.

Box 1 **Current situation of vaccine development targeting flaviviruses.**

There are currently no approved modalities for the treatment of flaviviral infections. Additionally, in case of secondary DENV infection with a different serotype of DENV, pre-existing antibodies (Abs) against DENV augments infection via antibody-dependent enhancement (ADE) [27,28]. Pre-existing Abs form complexes with viral particles, which bind to monocytes/macrophages via Fc receptors on the cell surface, resulting in enhanced infections in these cell types. In fact, most severe DENV cases are associated with secondary infection with a heterologous serotype of DENV [27,28]. Also, pre-existing Abs targeting DENV can augment the infection of ZIKV [29,30,31]. While vaccines targeting YFV and JEV are available, this complication of ADE makes it difficult to develop effective vaccines against infections with DENV and ZIKV. Therefore, the development of antiviral drugs targeting the “Achilles’ heel” of flaviviruses is strongly desired.

Flavivirus assembly follows the formation of replication complexes on the endoplasmic reticulum (ER) that consist of seven non-structural proteins and genomic RNA. The flavivirus genome is an approximately 11 kb, single-stranded, positive-sense RNA molecule encoding three structural proteins (core, prM, and envelope) and seven non-structural (NS) proteins. These viral proteins are translated from a single genomic RNA as a polyprotein, which is then cleaved by cellular proteases, viral proteases, and a signal peptidase. Thus, flavivirus replication depends on cellular enzymes and host intracellular organelles, such as secretary vesicles, the Golgi apparatus, and ER. Several lines of evidence suggest that flavivirus infection induces rearrangements of the ER membrane to generate vesicle packets that optimize RNA replication and virion assembly, shielding the viral components from the host immune response, such as interferon-mediated inhibition [32,33,34,35,36,37].

**Figure 2:**
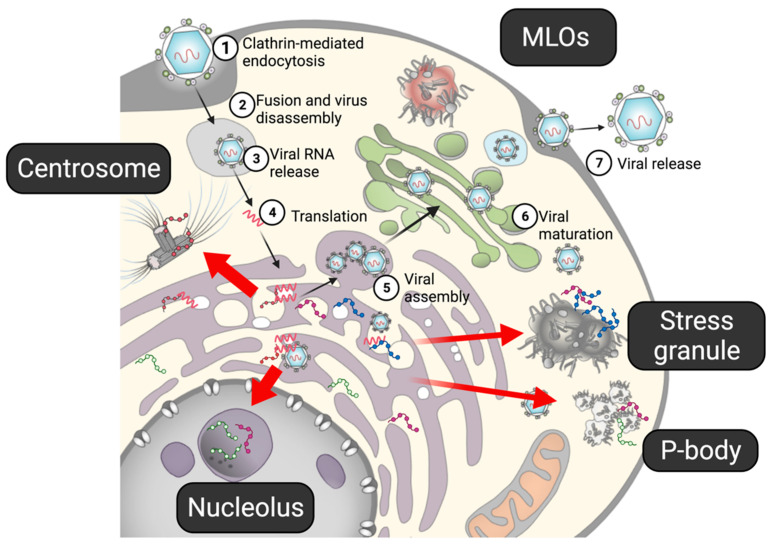
The life cycle of flavivirus. (**1**) After a flavivirus attaches to a specific receptor on a cell, it enters the cell via clathrin-mediated endocytosis. (**2,3**) Under the low-pH condition in the endosome, the viral capsid is disassembled, and viral RNA is released into the cell. (**4**) Viral RNA, acting as mRNA, is directly translated into viral proteins in the ER. The non-structural proteins form a replication complex to propagate viral RNA, while structural proteins form viral particles. (**5**) Viral assembly also occurs in the ER. (**6**) The prM on the immature viral particles is cleaved by furin in the Golgi apparatus. (**7**) Mature virions are released from the cell. Several viral proteins have been shown to interact with the MLOs. Flaviviral proteins have been shown to interact with MLOs (red arrows).

In addition to the vesicle pocket, flaviviral proteins are known to localize in MLOs, such as the core protein and NS5 in nucleoli, and the core and some NSs proteins in stress granules (SGs) (Figure 2). Mutations in the core protein abolish the protein’s nucleolar localization and reduce the infectivity of the virus [38], suggesting that the nucleolar localization of the core protein plays a critical role in viral replication. Nucleolar localization of the core protein is also observed in other flaviviruses, including WNV, DENV, and ZIKV [4], indicating a general function of the core protein in the nucleoli. Because many flaviviral proteins, including the core protein, have IDRs, they may be involved in LLPS. In this article, the properties of the disordered viral proteins obtained from genomic and proteomic analyses are reviewed. In addition, the potential interplay between the flaviviral proteins and MLOs, e.g., nucleoli and SGs, in the viral life cycle are discussed.

## 2. Disordered Regions in Viral Proteins

### 2.1. Viral Proteins Are Rich in Disordered Regions

An intrinsically disordered region (IDR), also referred to as intrinsically disordered protein (IDP), is a protein region with no particular secondary or tertiary structure. The lack of a rigid three-dimensional structure allows an IDR to interact with several partners simultaneously. IDRs possess a high content of charged residues and a low content of hydrophobic residues. The propensity for disorder of a given protein or peptide can be estimated from the amino acid sequence with the use of several algorithms, including IUPred (http://iupred.enzim.hu/index.html [39]), DisProt (https://disprot.org/ [40]), and PONDR (http://www.pondr.com/ [41]) (all of these sites were accessed on 21 July 2021). Bioinformatic analyses of genomic sequences from different kingdoms (i.e., bacteria, archaea, and eukaryotes) using these algorithms revealed relationships between the length and frequency of IDRs and complexity of the organism [42,43,44], as 7–30% of prokaryotic proteins and 45–50% of eukaryotic proteins contain disordered regions with >30 consecutive residues [42,43,45].

Quantification of IDRs in various viral proteomes revealed that viruses have the largest range of variation in the content of disordered residues ranging from 7.3% in human coronavirus NL63 to 77.3% in avian carcinoma virus [3]. Interestingly, there is a reverse relationship between the proteome size and the IDR content. The proteomes of small viruses with five or less proteins have 50% or more disordered residues: the greatest number of all species [3]. As the proteome size increases, the IDR content decreases to 20–40%. Another informatics study of the genome of 2278 viruses in 41 families revealed that the number of IDRs was correlated with the genome size [46]. Viral families with smaller genomes have higher contents of IDRs within five of the six main viral types (ssDNA, dsDNA, ssRNA(+), dsRNA, and retroviruses), with the exception of ssRNA(−), in which the proportion of IDRs increases with the genome size. This relationship between the proteome/genome size and the IDR content shows a clear contrast to that in eukaryotes, which have an IDR content as high as 40–50%, regardless of the proteome/genome size. The high IDR content of small viral proteomes seems to support the idea that viruses utilize IDRs/IDPs to increase flexibility and/or multi-functionality of individual proteins and gain broader adaptability to environmental changes from small amounts of genomic information, indicating that a high IDR content enables efficient usage of genomic information. Alternatively, IDR may play a critical role in the function of a minimum protein component that is necessary for virion assembly, but less in other accessory proteins. More structural information on viral proteins and bioinformatics analyses are required to test these possibilities.

### 2.2. Advantages of High IDR Content in Viral Strategies

The high IDR content of viral proteins implies that IDR is a general and essential strategy for efficient viral replication. There are several possible explanations for this. First, the lack of any particular three-dimensional structure allows for interactions with more than one partner molecule. In contrast to the stereospecific interactions between/among the solid surfaces of structured proteins, an IDR can interact with multiple solid surfaces with different structures (induced fit). In addition to this order–disorder interface, disorder–disorder interactions are also possible. Moreover, promiscuous and multivalent interactions can occur within a single type of IDR (if the polypeptide is polyampholyte), as well as among several different types of IDRs (especially between IDRs with opposite net charges). In some cases, such interactions induce LLPS (see later section). An IDR can also expand the variety of interactions by connecting two structured (functional) domains as a flexible linker, so as to increase the accessible surface area of the structured domain. These multi-interaction mechanisms mediated by IDRs confer functional advantages to viral proteins, which must interact with various host molecules, including proteins, membrane components, and nucleic acids, at different steps of the entire life cycle.

**Figure 3:**
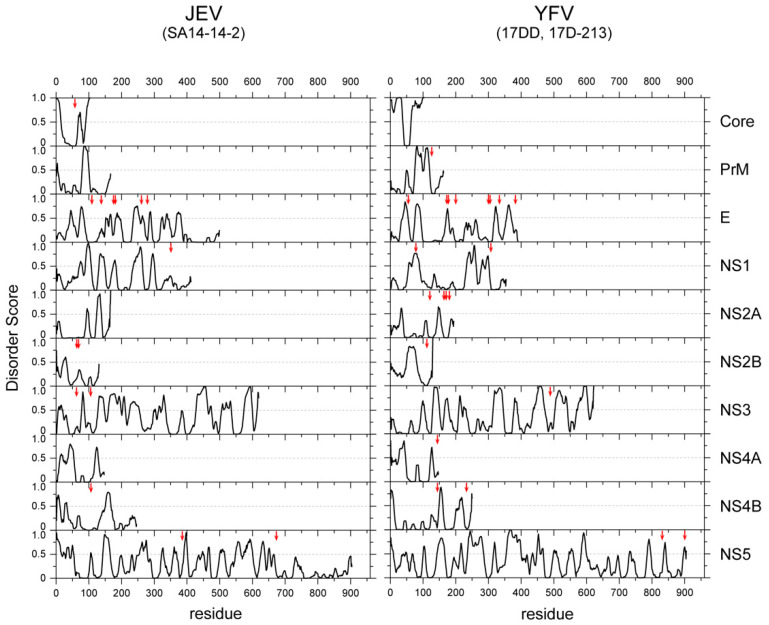
Amino acid substitutions occurred predominantly in the structured regions of the viral proteins of a vaccine strain. The IDR scores of all the proteins of JEV (**left**) and YFV (**right**) are plotted. The positions of the amino acid mutations found in the vaccine strains (SA14-14-2 for JEV [48] and 17D and 17DD-21-3 of YFV [49]) are indicated with vertical red arrows.

Another advantage of the viral IDR is the high mutation rate. The lack of structural constraints may help to tolerate high mutation rates and promote faster adaptation to environmental changes. Viruses must survive both within the host cells and the host environments (extracellular spaces) by adapting to a variety of environmental factors, including antiviral drugs and the host immune response. Generally, the viral genome exhibits an extremely higher mutation rate (10^−5^–10^−3^ for RNA viruses and 10^−8^–10^−5^ for DNA viruses per position per generation) than the bacterial and eukaryotic genomes (10^−9^ on average) [47]. Although the accuracy of polymerases and the replication machinery is the major driving force of such high mutation rates, the structural constraints of translated polypeptides also contribute to this. Furthermore, overlapping reading frames, which can be found in many viral genomes, also accelerate the evolution of viral proteins. It is intriguing that mutations to flaviviral proteins, which abolish viral infectivity, are mostly found in structured domains, rather than the IDRs (Figure 3), implying that IDRs are more tolerant to mutations than structured domains.

### 2.3. RNA Chaperone Activity of Viral IDRs

RNA chaperoning activity is one of the distinct and unique functions of IDRs. RNA chaperones exist in various cell types, including prokaryotes and eukaryotes, and play pivotal roles in many biological processes, such as transcription, mRNA export, splicing, and ribosome biogenesis. An RNA chaperone binds to, partially unfolds, and finally refolds misfolded RNA into a more stable conformation. Because RNA is prone to post-transcriptional misfolding [50], chaperone activity is necessary for proper folding and function of cellular RNA. Many RNA viruses and retroviruses also have proteins with RNA chaperone activity. For example, the nucleocapsid protein NCp7 of human immunodeficiency virus type I (HIV-1) has RNA chaperone activity, which is necessary for maturation, reverse transcription, and integration [51,52,53] (see later section).

The results of a proteomic study demonstrated that known RNA chaperones, as well as protein chaperones, contain high percentages of IDRs (highest of all proteins groups) [54]. In the case of RNA chaperones, promiscuous interactions between basic residues within the IDR and RNA promotes annealing of two complementary sequences or refolding of misfolded RNA molecules [55]. Although the molecular mechanism of the chaperone activity of IDRs remains unclear, Tompa et al. proposed an “entropy exchange model,” where the partial order–disorder transition of a chaperone IDR is coupled to the unfolding–refolding conversion of the substrate to a more stable conformation [54]. This shows a clear contrast to the typical catalytic activities of structured enzymes of stereospecific interactions between the catalytic pocket of the enzyme and the substrate, which are critical for the reaction. Notably, the first protein enzyme appearing in the RNA world could have been an RNA chaperone [56].

The RNA chaperones of RNA viruses and retroviruses also contain significant numbers of IDRs. For example, the NCp7 protein of HIV-1, a short polypeptide of 55 amino acids, is derived from the Gag polyprotein and contains two CCHC-type zinc fingers [57]. Disorder prediction, as well as NMR solution structure, revealed that zinc fingers have relatively defined structures, although other flanking sequences are disordered in the absence of RNA [58,59,60]. The nucleocapsid protein recognizes packaging sequences within the viral RNA genome and promotes efficient packaging into a virion. Due to its basic amino acid composition, nucleocapsid protein binds to RNA in a sequence-independent manner and functions as an RNA chaperone to promote several RNA-dependent steps in the HIV life cycle, such as annealing of the tRNA primer, stimulating integration [61], and promoting DNA strand exchange reactions during reverse transcription [62,63,64]. The functional roles of IDRs of other HIV-1 accessory proteins are reviewed in a previous article [60].

**Figure 4:**
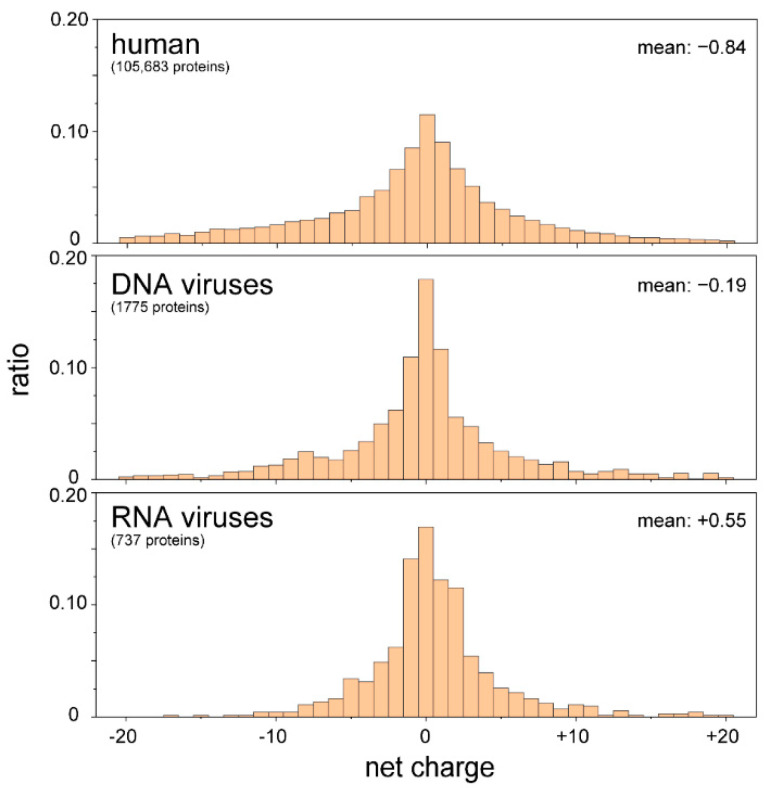
IDRs of RNA viruses are positively charged. The amino acid sequences of IDRs were extracted from proteomic databases of the human, DNA viruses, and RNA viruses. The net charges of the IDRs of individual proteins were calculated and are summarized as a histogram. IDRs carrying more than 21 net charges (either negative or positive) were omitted. There were significant differences between human and RNA viruses, as well as DNA and RNA viruses, with *p*-values of less than 0.01 (*t*-test).

The core proteins of viruses in the family *Flaviviridae* (i.e., hepatitis C virus (HCV), GB virus B, West Nile virus (WNV), and bovine viral diarrhea virus (BVDV)-1) are also required for packaging the RNA genome and protecting it from the host immune response. The N-terminal IDR has RNA chaperone activity [65,66,67,68]. It can be speculated that RNA viruses use the IDRs to control the conformation of the RNA genome to facilitate replication, reverse transcription (in the case of retroviruses), and packing. Our proteomic analyses demonstrated that the IDRs of RNA viruses are rich in basic amino acids, whereas human IDRs and those of DNA viruses are slightly negative (Figure 4). These results imply that RNA viruses use basic IDRs to interact with and promote proper folding of genomic RNA in the host cell. In the following section, possible roles of IDRs/IDPs in the life cycles of flaviviruses are discussed.

## 3. Impact of Intracellular MLOs on the Flaviviral Life Cycle

### 3.1. Flaviviral Proteins in Nucleolus

A number of viral proteins from all viral classes, including BVDV, influenza, HIV, and adeno-associated virus, localize in the nucleolus and interact with nucleolar proteins [69,70,71]. It is not surprising that viruses that replicate in the nucleus (many DNA viruses and some ssRNA(−) viruses and retroviruses) interact with the nucleolus. However, of particular interest is the fact that many RNA viruses that replicate and package the virion outside of the host cell nucleus also interact with the nucleolus.

Notably, core proteins of several *Flaviviridae* viruses are targeted to the nucleoli [72]. The core protein of *Flaviviridae* virus is an icosahedral capsid dimer that is approximately 11–14 kDa in size [73,74,75,76]. The mature core protein is composed of approximately 105 residues. A monomer is composed of an N-terminal IDR carrying positively charged residues, multiple α-helices (α1-4), and a hydrophobic C-terminal domain as well as the transmembrane α5 helix (also termed as a capsid anchor), which works as a signal peptide to ensure the topology of the membrane protein [74,76,77,78].

As described above, the core protein is responsible for the packaging of genomic RNA into the viral particle [74,79]. Since the N- and C-termini contain nucleic acid-binding sites [76,80], mutations to the N-terminal residues impair viral particle formation of DENV [81] and tick-borne encephalitis virus [82]. Mutations to the hydrophobic surface residues in the middle α-helices impairs viral propagation [83]. Moreover, previous studies have reported that the core protein interacts with cellular proteins to induce apoptosis [84].

As mention above, flaviviral core proteins are targeted to the nucleoli. The WNV core protein translocates in the nucleus and interacts with the nucleolar protein DDX56 [85]. The DDX56 is involved in the infectivity of WNV, suggesting that WNV utilizes interaction with DDX56 for optimal replication.

A similar observation was reported with JEV core protein. Substitutions of Gly42 and Pro43 of JEV core protein to Ala (GP42/43AA) abolished translocation of the core protein to the nucleus. Recombinant JEV possessing a core protein with GP42/43AA substitutions impaired viral growth both in vitro and in vivo, suggesting that localization of the core protein to the nucleoli is critical to the life cycle of JEV [38]. This defect can be, at least partly, explained by a prvious finding that appropriate localization of JEV core protein is critical for assembly of viral particles and viral release from the infected host cell [86]. What are the cellular partner(s) interacting with JEV core protein? The JEV core protein interacts with the nucleolar protein B23 (nucleophosmin), which is translocated from the nucleolus to the cytoplasm during infection and is involved in viral replication [72]. The interaction between core protein and B23 has been observed with other flaviviruses. Fluorescence loss in photobleaching-mediated imaging analysis revealed that the core protein of DENV-2 affects the mobility of B23 [87]. Additionally, the core proteins of all serotypes of DENV (DENV1–4) were shown to target the granular component of the nucleolus, which is formed by B23. Most importantly, both B23 and flaviviral core proteins display LLPS [87].

Viruses either (i) target their own proteins to the nucleoli to modify nucleolar and cellular functions, (ii) utilize some of the nucleolar components (proteins and RNAs) to mediate reactions or roles necessary for the viral life cycle, or (iii) use the nucleolar “environment” to evade sensing/recognition by the host cell immune response. Since the core protein binds RNA with broad sequence specificity [65,88], it is highly probable that it also binds to the host cellular RNA in the nucleoli, such as rRNA and small nucleolar RNA. Such RNA may stabilize the structure of the core protein in the nucleolus in preparation for future functions. It is tempting to test how a mutation to the JEV core protein, which renders it incapable of translocating to the nucleolus, changes interactions with various classes of RNA. Additionally, disruption of the critical communication between the core protein and cellular RNA presents an attractive target for the development of antivirals.

### 3.2. Assembly/Dissolution of SGs during Flaviviral Infection

SGs are also an important interface between the host cell and viral functions. SGs are formed as a consequence of shut down of translation and release of mRNA from polysomes, when cells are exposed to stress (oxidative, heat, osmotic, or infection). SGs contain mRNA and RNA-binding proteins. Several IDR-containing proteins such as FUS RNA-binding protein and heterogeneous nuclear ribonucleoprotein A1 localize to SGs [89,90]. In addition, the IDR-rich protein G3BP plays a critical role in SG formation [91]. Most importantly, these proteins have been shown to undergo phase separation in vitro.

It has been shown that JEV inhibits the arsenite-induced SGs formation in infected cells. This activity depends on the interaction between the JEV core protein and cellular Caprin-1 protein [12], a SG-associated protein that binds to the G3BP and regulates G3BP-mediated SG formation. In *CAPRIN1*-depleted Huh7 cells, infection of JEV failed to inhibit arsenite-induced SG formation and resulted in impaired viral propagation. Furthermore, mutagenesis study of the residues essential for interactions with Caprin-1 (substitution of Lys97/Arg98 of core protein to Ala; KR97/98AA mutant) also abrogated inhibition of arsenite-induced SG formation. Furthermore, this core mutant showed impaired viral replication in vitro and reduced pathogenicity in infected mice. These data suggest that the JEV core protein specifically interacts with Caprin-1 to inhibit SG formation for efficient viral propagation [12].

A similar observation has been reported with other flaviviruses. WNV and DENV-2 infection also inhibits arsenite-induced SG formation [13]. Formation of SGs requires phosphorylation of eukaryotic translation initiation factor 2α (EIF2A) by several kinases, including protein kinase R (PKR). Unlike WNV strains NY99 and Eg101, infection with WNV strain W956IC naturally induces PKR-dependent SG formation [92]. A recombinant WNV of strains Eg101 and W956IC revealed that non-structural proteins (NS1, NS3, NS4A, NS4B, and NS5) are involved in the formation of SGs by strain W956IC. Comparative analyses suggested that the higher activity of viral RNA synthesis in strain W956IC exposed viral dsRNA in the cytosol, which is recognized by PKR to stimulate SG formation.

Similarly, infection with ZIKV inhibited arsenite-induced SG formation by preventing phosphorylation of EIF2A [93,94]. Interestingly, ZIKV infection did not inhibit EIF2A-independent SG formation. Previous studies demonstrated that EIF2A is de-phosphorylated by protein phosphatase 1A with the regulatory subunit growth arrest and DNA damage-inducible protein 34 (GADD34) [94]. ZIKV infection upregulated GADD34 expression, implying that de-phosphorylation of EIF2A is promoted in ZIKV-infected cells. Supporting this scenario, treatment with Salubrinal, which inhibits de-phosphorylation of EIF2A, enhanced arsenite-induced SG formation and reduced viral propagation in a dose-dependent manner [94]. These data suggest that ZIKV infection upregulates GADD34 to de-phosphorylate EIF2A, inhibiting SG formation. In contrast to this report, Hou et al. showed that ZIKV infection inhibited both EIF2A-dependent and -independent SG formation [95].

Furthermore, infection with the Usutu virus, a member of the genus *Flavivirus*, also inhibited arsenite-induced EIF2A phosphorylation and SG formation. Activation of antioxidant molecules is involved in the inhibition of arsenite-induced SG formation [96]. WNV infection increased the levels of glutathione, an abundant intracellular antioxidant molecule, through activating transcription factor 4 (ATF4) and NF-E2-related factor 2 (NRF2). Knockdown of ATF4 or NRF2 cancelled the inhibition of arsenite-induced SG formation. However, the contribution of viral proteins to the inhibition SG formation remains unclear [97].

In conclusion, the inhibition of SG formation via several machineries (interaction with Caprin-1, de-phosphorylation of EIF2A, etc.) can be a shared strategy for flaviviruses to optimize viral replication.

### 3.3. Functional Interaction between Viral Proteins and SGs

Many efforts have been made to elucidate the biological role of SG formation in viral infection. One of the biggest questions is whether SGs are antiviral or proviral. As described above, the core protein is responsible for suppression of SG formation in flavivirus-infected cells. Additionally, non-coding RNA of DENV-2 targets and antagonizes the function of G3BP1, G3BP2, and Caprin-1 [98]. Previous studies have demonstrated that G3BP1 binds to double-stranded RNA and inhibits replication of RNA virus by positively regulating a RIG-I-mediated cellular antiviral response [99,100]. Therefore, it is tempting to speculate that flaviviruses abolish SG formation to avoid sensing by RIG-I. Similarly, the alphaviral nsP3 protein inhibits SG formation by translocating G3BP1 from SGs [101,102]. Conversely, G3BP1 depletion attenuates replication of alphaviruses [103], indicating that G3BP1 is crucial for amplification of the viral RNA genome. Furthermore, G3BP1 and G3BP2 regulate translation of interferon-induced transmembrane 1, 2, and 3 [104], which are major antiviral proteins. These findings suggest that SG formation is delicately balanced during the replication of RNA viruses.

Another intriguing interface between viral IDRs and SGs is the regulation by multisite phosphorylation. The dynamic assembly/disassembly of SGs is regulated by phosphorylation of the IDR of G3BP1 by casein kinase 2 [105]. The IDR of the NS5A protein of HCV is also hyper-phosphorylated by casein kinase I or II [106,107]. The NS5 of flaviviruses is a conserved-phospho-protein [108,109,110,111] that is reportedly phosphorylated by casein kinase I [112] or protein kinase G [113]. In general, phosphorylation predominantly occurs to the IDRs [114,115,116], which regulates phase separation and intracellular RNA granule dynamics [117,118,119,120]. Although the detailed mechanism of phosphorylation-dependent regulation of phase separation has not yet been clarified, the segregation of charged residues along the polypeptide chain (linear distribution of oppositely charged blocks) plays a critical role in enhancing phase separation [121,122,123,124]. Indeed, the introduction of multiple phosphate groups to the IDR of HCV NS5A changes the charge distribution. Therefore, it is highly probable that the IDRs of not only SG proteins, but also many viral proteins, undergo phosphorylation at multiple sites to regulate the formation/dissolution of SGs and other MLOs.

## 4. Closing Remarks and Future Directions

In this review, the basic characteristics of IDR as well as the complex interplay between viral proteins and MLOs are summarized. There must be selective pressure for RNA viruses to maintain and/or evolve the IDRs of viral proteins to optimize replication, adapt to new host species, and avoid stimulation of host immune sensors. Recent advances in research tools and understanding of LLPS has allowed for the bridging of the traditional fields of biophysics and virology, thereby creating a new field to understand the complex and sophisticated events during viral replication.

Recent studies have reported that the nucleoprotein of severe acute respiratory syndrome coronavirus 2 (SARS-CoV-2) induces LLPS in the presence of viral RNA [125,126]. The nucleoprotein of SARS-CoV-2 co-localizes with G3BP, a marker of SGs. Once phosphorylated, the serine/arginine-rich region of the nucleoprotein interacts with RNA. LLPS of the nucleoprotein and RNA can recruit non-structural protein (NSP) 7, NSP8, and NSP12, suggesting that LLPS of the nucleoprotein is required for the condensation of the viral replication machinery [127]. Importantly, the (−)-gallocatechin gallate (GCG), a polyphenol from green tea, was found to disrupt the interaction between the nucleoprotein and RNA and inhibit the formation of LLPS of the nucleoprotein [128]. Treatment with GCG suppressed viral RNA replication of SARS-CoV-2, suggesting that the LLPS of SARS-CoV-2 can be a therapeutic target for COVID-19 [128]. In addition, the nucleoprotein and phosphoprotein of measles virus (MeV) are also reported to form MLOs through LLPS, which are then co-localized with RNA and contribute to efficient viral replication [129]. These data imply that MLOs formed by the viral replication machinery might be constructed by LLPS, similar to MeV [130]. The targeting of LLPS, as shown by GCG of SARS-CoV-2, has potential as an antiviral strategy, although further investigations are needed to determine the importance of LLPS on the life cycles of other RNA viruses and to develop specific inhibitors against viral LLPS.

## Figures and Tables

**Table 1:** Interactions of viral proteins with MLOs.

MLOs	Viral Family	Viral Protein (Virus)	References
Nucleolus	*Flaviviridae*	Core (JEV, DENV, WNV, ZIKV)	[4]
		NS5 (DENV), NS5B (HCV)	[5,6]
	*Nidovirales* *(Tobaniviridae, * *Arteriviridae, and* *Coronaviridae)*	N (ToV, PEDV, SARS-CoV, IBV, PRRSV)	[7,8]
		NSP3B (FCoV, SARS-CoV)	[9,10]
	*Picornaviridae*	2A (EMCV)	[11]
SG	*Flaviviridae*	Core (JEV)	[12]
		NSs (WNV, DENV)	[13]
	*Picornaviridae*	2A (PV, EV71)	[14,15]
	*Retroviridae*	Gag (HIV-1)	[16]
		Tax (HTLV-1)	[17]
P-body	*Flaviviridae*	Core, NS5A? (HCV)	[18]
	*Reoviridae*	NSP1 (RV)	[19]
	*Picornaviridae*	3C (PV)	[20]
Centrosome	*Flaviviridae*	NS5 (ZIKV)	[21]
	*Retroviridae*	Gag (HIV-1, FV)	[22]
		Tax (HTLV-1)	[23]

Abbreviations; DENV—dengue virus, EMCV—encephalomyocarditis virus, EV71—enterovirus71, FCoV—feline coronavirus, FV—foamy virus, HCV—hepatitis C virus, HIV-1—human immunodeficiency virus type I, HTLV-1—human T-lymphotropic virus type I, IBV—infectious bronchitis virus or avian coronavirus, JEV—Japanese encephalitis virus, PEDV—porcine epidemic diarrhea virus, PRRSV—betaarterivirus suid 1, formerly porcine reproductive and respiratory syndrome virus, PV—poliovirus, RV—rotavirus, SARS-CoV—severe acute respiratory syndrome-related coronavirus, ToV—Torovirus, WNV—West Nile virus, YFV—yellow fever virus, ZIKV—Zika virus.

## Data Availability

Intrinsic disordered residues were predicted with the IUPred2A program, by using the short disorder option (https://academic.oup.com/nar/article/46/W1/W329/5026265). The residues with IUPred score >0.5 were regarded as disordered residues. The protein sequences examined and their metadata were obtained from the NCBI virus database (https://www.ncbi.nlm.nih.gov/labs/virus/vssi/#/) and the NCBI protein database (https://www.ncbi.nlm.nih.gov/protein/) (all of these sites were accessed on 3 May 2021). We here focused on the REFSEQ sequences of proteins for viruses whose hosts are human and human proteins.
